# Optical, electrochemical and photophysical analyses of heteroleptic luminescent Ln(iii) complexes for lighting applications†

**DOI:** 10.1039/d3ra00214d

**Published:** 2023-03-20

**Authors:** Anjli Hooda, Devender Singh, Anuj Dalal, Kapeesha Nehra, Sumit Kumar, Rajender Singh Malik, Ramesh Kumar, Parvin Kumar

**Affiliations:** a Department of Chemistry, Maharshi Dayanand University Rohtak 124001 India devjakhar@gmail.com; b Department of Chemistry, DCR University of Science & Technology Murthal Haryana 131039 India; c Department of Chemistry, Kurukshetra University Kurukshetra 136119 Haryana India

## Abstract

A series of lanthanide complexes have been synthesized with fluorinated 1,3-diketones and heteroaromatic ancillary moieties. Spectroscopic studies reveal the attachment of the respective lanthanide ion to the oxygen site of β-diketone and nitrogen site of auxiliary moieties. The conducting behavior of the complexes is proposed by their optical energy gaps which lie in the range of semiconductors. The emission profiles of the lanthanide complexes demonstrate red and green luminescence owing to the distinctive transitions of Sm^3+^ and Tb^3+^ ions, respectively. Energy transfer *via* antenna effect clearly reveals the effective transfer of energy from the chromophoric moiety to the Ln^3+^ ion. The prepared conducting and luminescent Ln(iii) complexes might be employed as the emitting component in designing OLEDs.

## Abstract 2

A series of lanthanide complexes have been synthesized with fluorinated 1,3-diketones and heteroaromatic ancillary moieties.

## Graphical abstract

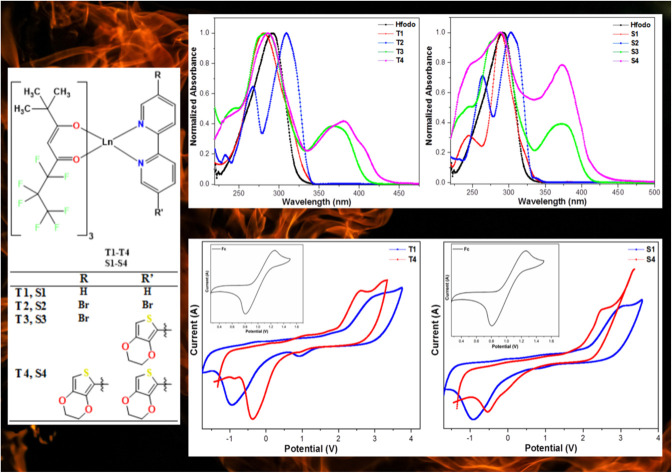

## 1 Introduction

The coordination compounds of lanthanide (Ln) ions having *a* +3 oxidation state exhibit exciting photo-luminescent features which are promising for their vast range of applicability in diverse fields comprising optical amplifiers,^1^ sensors,^2^ lasers,^3^ fluorescent probes,^4,5^ single molecule magnets (SMM)^6,7^ and OLEDs.^8,9^ These Ln complexes have narrow monochromatic emission peaks due to intraconfigurational transitions which belong to the 4f subshell, large Stokes displacement, long emissive state life-time, and good quantum yield.^10,11^ However, the solitary metal ions cannot be utilized as luminescent materials due to parity prohibited transition and low molar absorptivity in the ultraviolet-visible (UV-vis) region. The above outcomes demonstrate the weak and low luminescent efficiency of lanthanides.^12,13^ Thus, trivalent ions typically form coordinated complexes with organic ligands. These organic moieties possess strong absorption in the UV-vis region and upon coordination with the metal ion transfer the absorbed energy to it. Therefore the organic moiety proficiently sensitizes and improves the photoluminescence efficiency of 4f ions. This mechanism is titled as “antenna effect”.^14–16^ Existence of solvent units in lanthanide complexes are greatly unsuitable on account of highly energized stretching vibrators of hydroxyl and amino modes which leads to luminescence quenching.^17,18^ It can prominently restrict the practicability of ternary complexes as luminescent material. The high energetic oscillators in chromophoric moieties are also reasonable to quench the luminescence phenomenon in lanthanide ions.^19,20^ Thus, the substitution of C–H by less energetic C–F bond minimizes the luminescence quenching. The additional assistance of fluorination is the heavy atom effect, which can increase the efficiency of intersystem crossing (ISC) from singlet to triplet level of ligand.^21–23^ Neutral ligands can substitute the solvent molecules and form coordinatively saturated lanthanide complexes *via* hard donor sites *i.e.* nitrogen and oxygen. These ligands have provided rigidity and thermal stability to the complexes. Asymmetric coordinating environment around central ion has also enhanced the luminescence characteristics.^24^

Lanthanide ions such as europium, samarium and terbium have exhibit bright red, orange and green emission, respectively in visible-range of the electro-magnetic spectrum (EMS). Now we have taken Tb^3+^ and Sm^3+^ ions for synthesis of ternary complexes. Tb^3+^ ion exhibits green emission which is the component of red-green-blue system accredited to transition of ^5^D_4_ → ^7^F_5_ and situated about emissive wavelength of 545 nm.^25,26^ The ternary samarium complexes show red luminescence due to ^4^G_5/2_ → ^6^H_9/2_ (648 nm) transition.^27,28^

Here, we have reported eight Ln(iii) complexes based on fluorinated di-ketone 2,2-dimethyl-6,6,7,7,8,8,8-heptafluoro-3,5-octanedione (Hfodo) and N-donor ancillary units which are 2,2′-bipyridine [Bpy], 5,5′-dibromo-2,2′-bipyridine [DBr], 5-bromo-5′-(3,4-(ethylenedioxy)thien-2-yl)-2,2′-bipyridine [MD] and 5,5′-bis(3,4-(ethylenedioxy)thien-2-yl)-2,2′-bipyridine [DD]. The main aim of our work is to assess the electronic impact of substituents on the optoelectronic and photophysical characteristics of synthesized complexes. The complexes Tb(Hfodo)_3_Bpy (T1), Tb(Hfodo)_3_DBr (T2), Tb(Hfodo)_3_MD (T3), Tb(Hfodo)_3_DD (T4), Sm(Hfodo)_3_Bpy (S1), Sm(Hfodo)_3_DBr (S2), Sm(Hfodo)_3_MD (S3) and Sm(Hfodo)_3_DD (S4) have been reported. These lanthanide complexes were examined by elemental investigation, spectroscopically, thermal gravimetric and electro-chemical technique. Complexes were colorimetrically analyzed by using the results of photoluminescence emission.

## 2 Experimental

### 2.1 Chemicals and instrumentation

The chemicals comprising Bpy, Hfodo, TbCl_3_·6H_2_O and SmCl_3_·6H_2_O were bought from Sigma Aldrich (SA). The reagent such as 25% NH_4_OH solution and solvents *viz.,* methyl carbinol and hexyl hydride has been utilized directly. The ligands namely DBr, MD and DD have been synthesized in lab.^29^ The composition of CHN in lanthanide complexes was inspected *via* 2400-CHN Analyzer. The FTIR and proton NMR spectral data was measured on a PerkinElmer 400 FTIR spectrophotometer and FT NMR spectrometer respectively. The proton NMR signals were obtained in CDCl_3_ and [(CH_3_)_4_Si] as reference. Absorption and electrochemical analyses were performed on as respective instrument such as Shimadzu UV VIS 2450 and Potentiostat-4000. PL spectral information was measured on a Horiba Fluorolog 3. Thermal-gravimetric [TG] and differential-thermal-gravimetric [DTG] patterns were obtained under nitrogen atmosphere on a Hitachi Simultaneous Thermogravimetric Analyzer-7300.

### 2.2 Synthesis

**Fig. 1:**
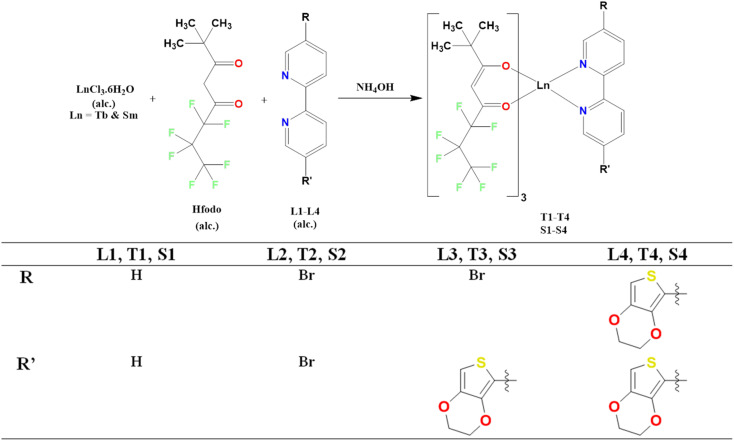
Schematic representation of ternary lanthanide complexes.

Fig. 1 represents the schematic way for synthesizing lanthanide complexes.^30–33^ Ln(iii) complexes were made by adding 6.48 mmol of 25% NH_4_OH to 6.48 mmol of Hfodo in 5 mL of methyl carbinol. Beaker was retained undisturbed and closed till the smell of ammonia evaporates. Then, 2.16 mmol of alcoholic solution of lanthanide (respective samarium and terbium) chloride hexahydrate and substituted 2,2′-bipyridine ligands were dispensed to ammoniated solution of Hfodo. The resulting mixture (pH = 6–7) was agitated for 12 hours. After that, leaves the beaker for slow disappearance of solvent. Methyl carbinol and hexyl hydride (2–3 times) were employed to wash the solid residue.

## 3 Results and discussion

### 3.1 Primary interpretation

The basic (CHN) compositional data as well as preliminary findings such as appearance and quantity of the complexes is listed in Table 1. The preliminary findings confirm that the estimated outcomes are accordant with the expected data. Furthermore, the findings illustrate that lanthanide compounds are formed in precise stoichiometric proportions. The synthesized lanthanide complexes are solvable in dichloromethane and dimethylsulfoxide.

**Table 1:** CHN data of lanthanide complexes

Complex	Color	Yield (%)	C% obs.(calcd.)	H% obs.(calcd.)	N% obs.(calcd.)
T1	White	40	39.85 (39.91)	3.41 (3.43)	2.40 (2.33)
T2	White	33	35.37 (35.29)	2.91 (2.89)	1.99 (2.06)
T3	Yellow	48	38.76 (38.83)	3.17 (3.12)	1.95 (1.97)
T4	Yellow	37	42.05 (42.09)	3.39 (3.33)	1.92 (1.89)
S1	White	35	40.38 (40.30)	3.17 (3.21)	2.29 (2.35)
S2	White	45	35.63 (35.59)	2.71 (2.69)	2.01 (2.08)
S3	Yellow	50	39.05 (39.15)	2.99 (2.93)	2.03 (1.99)
S4	Yellow	39	42.39 (42.42)	3.19 (3.15)	1.89 (1.90)

### 3.2 IR and ^1^H-NMR spectra evaluation

FTIR spectral information of Ln(iii) complexes are mentioned in Table 2. No band at 3300 cm^−1^ indicates that water molecules do not exist in the complexes. Stretching vibrations of =C–H unit are liable for the peak at 3060 cm^−1^. The FTIR spectrum of diketone ligand reveal a prominent peak due to C=O group which is moved to the lesser frequency after complexation. This suggests the attachment of the diketone to metal ion *via* oxygen.^34,35^ The finding of peaks at about 1070–1100 cm^−1^ correspondent to C–Br unit existed in T2, T3, S2 and S3. In FTIR profiles of some complexes (T3, T4, S3 and S4) two peaks in scope of 688–722 cm^−1^ are present due to C–S–C bond.^29^ The peaks around 455–475 cm^−1^ (Ln–O) and 531–538 cm^−1^ (Ln–N), confirms the involvement of Hfodo and ancillary moieties with the Ln^3+^ ion.^36–38^

**Table 2:** FTIR spectral information (in cm ^−1^) of lanthanide complexes

Complex	ν_(Ln–O)_	ν_(Ln–N)_	ν_(C_–_S_–_C)_	ν_(C_–_Br)_	ν_(C_–_N)_	ν_(C_–_F)_	ν_(C=C)_	ν_(C=N)_	ν_(C=O)_	ν_(_=_C_–_H, –C–H)_
T1	466	530	—	—	1125	1347	1464	1542	1605	3072, 2970
T2	458	535	—	1086	1123	1356	1456	1539	1611	3077, 2967
T3	478	538	722, 690	1073	1122	1346	1474	1538	1620	3075, 2970
T4	472	534	718, 688	—	1120	1350	1468	1547	1616	3075, 2972
S1	470	532	—	—	1122	1349	1460	1540	1615	3051, 2977
S2	457	538	—	1100	1123	1356	1456	1543	1620	3049, 2974
S3	477	537	722, 689	1102	1121	1346	1473	1539	1619	3055, 2972
S4	472	535	719, 692	—	1124	1354	1466	1542	1618	3052, 2975

Table 3 lists^1^H-NMR data of prepared complexes and 1,3-diketone. In their lanthanide complexes, the methine proton (=C–H) signal of Hfodo is displaced towards the lower chemical shift. The paramagnetic property of terbium and samarium ions causes noticeable alterations in the ^1^H-NMR spectrum data of ligands.^39–41^ The paramagnetic nature of Tb^3+^ greatly affects the position of signals of auxiliary units. Fig. S1–S16† represents IR and NMR spectral profiles of ternary terbium and samarium complexes respectively.

**Table 3:** ^1^H-NMR data (ppm) of prepared complexes

Complex	Peaks due to Hfodo	Peaks due to neutral ligands
Hfodo	14.82 (–OH), 5.62 (methine), 3.56 (–CH_2_), 1.29 (–C(CH_3_)_3_)	—
T1	116.08 (3H, methine –CH), 1.24–0.93 (27H, –CH_3_)	−9.45 (2H), −21.11 (2H), −30.05 (2H), −45.60 (2H)
T2	121.19 (3H, methine –CH), 1.40–0.69 (27H, –CH_3_)	−2.07 (2H), −15.85 (2H), −25.01 (2H)
T3	118.48 (3H, methine –CH), 1.28–0.84 (27H, –CH_3_)	−3.40 (2H), −4.72 (2H), −9.58 (2H), −12.16 (2H), −13.08 (1H), −16.83 (1H), −26.15 (1H)
T4	118.39 (3H, methine –CH), 1.33–0.84 (27H, –CH_3_)	−3.38 (2H), −4.74 (4H), −5.68 (2H), −6.67 (2H), −9.59 (1H), −117.1–13.19 (2H), −17.01 (1H), −18.70 (1H), −26.24 (1H)
S1	6.89 (3H, methine –CH), 1.06 (27H, –CH_3_)	7.66 (2H), 7.59 (2H), 7.46 (2H), 7.05 (2H)
S2	6.86 (3H, methine –CH), 1.08 (27H, –CH_3_)	8.71 (2H), 8.29 (2H), 7.93 (2H)
S3	6.94 (3H, methine –CH), 1.04 (27H, –CH_3_)	8.81 (1H), 8.37 (1H), 8.08 (2H), 7.55 (1H), 7.39 (1H), 6.50 (1H), 4.96 (4H)
S4	6.96 (3H, methine –CH), 1.06 (27H, –CH_3_)	7.95 (2H), 7.59 (2H), 6.75 (2H), 6.32 (2H), 4.32 (4H), 4.13 (8H)

### 3.3 Absorption spectral study

**Fig. 2:**
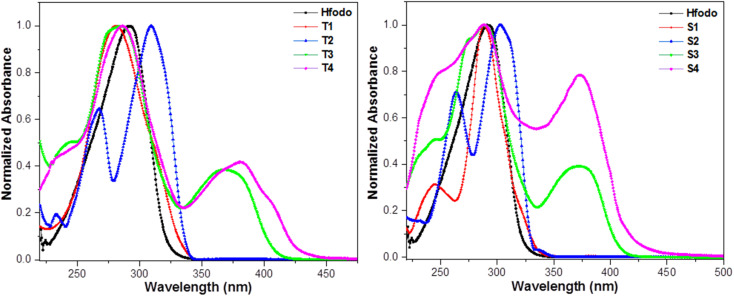
Absorption plots of synthesized Hfodo and Ln(iii) complexes.

At room temperature, UV–visible absorption spectra of Ln(iii) complexes were recorded in dichloromethane (DCM) solvent (10^−5^ M). Fig. 2 exhibit the absorption patterns of Hfodo and Ln(iii) complexes. The spectral profiles of synthesized complexes specify a band in 280–380 nm associated with the π–π* transitions of chelated moieties. Bathochromic shift is observed with change in ancillary moieties from Bpy to DD. Bands due to free units get displaced after complexation with metal ion.^42,43^ With the assistance of spectral profiles, optical band-gap (*E*_g_) was designed (eqn (1)).

1 *αℏν* = *α*(*ℏν* − *E*_g_)^*n*^

**Fig. 3:**
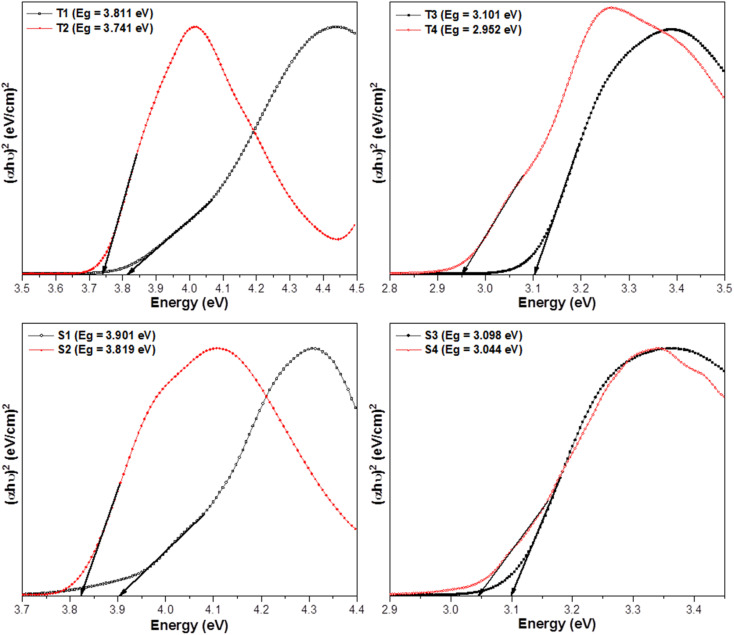
Tauc's profiles of prepared complexes.

The above relation includes absorption co-efficient (*α*); energy of photon (*ℏν*) and optical parameter (*n*).^44,45^*E*_g_ was determined by extrapolating a line to (*αhυ*)^2^ = 0 as given in Fig. 3. *E*_g_ declines in a particular series *i.e.* from T1 to T4 and S1–S4 suggesting the increase in conjugation. The band gap value lies in the range of semiconducting materials.

### 3.4 Thermal gravimetric study

**Fig. 4:**
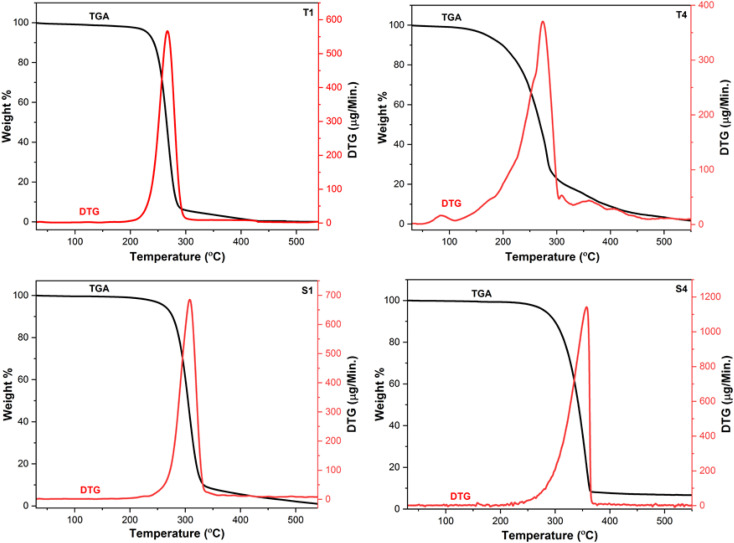
Thermograms of T1, T4, S1 and S4.

The thermograms of Ln(iii) complexes were collected in order to measure their thermal strength. The thermograms of T1, T4, S1 and S4 are thoroughly examined here because the disintegration tendencies of all the prepared complexes are quite comparable. Fig. 4 displays the thermogram of above mentioned complexes which demonstrates the single step disintegration. The unavailability of spectral variation up to 120 °C suggests the anhydrous nature of prepared compounds. T1 shows 85.23% (calcd: 86.79%) mass loss in temperature range of 213–330 °C while T4 exhibit 85.74% (calcd: 89.30%) mass loss in 127–368 °C due to the removal of ligands attached to Tb(iii) ion. In case of S1 and S4, huge mass loss of 89.67% (calcd: 87.42%) and 90.15% (calcd: 89.82%) in 225–343 °C and 246–366 °C is attributed to removal of three Hfodo and single neutral unit respectively. The residual product formed after decomposition was due to oxides of terbium and samarium.^46–48^ The peaks in DTG curves at 267 °C (T1), 275 °C (T4), 308 °C (S1) and 358 °C (S4) support their decomposition pattern.

### 3.5 Electrochemical study

**Fig. 5:**
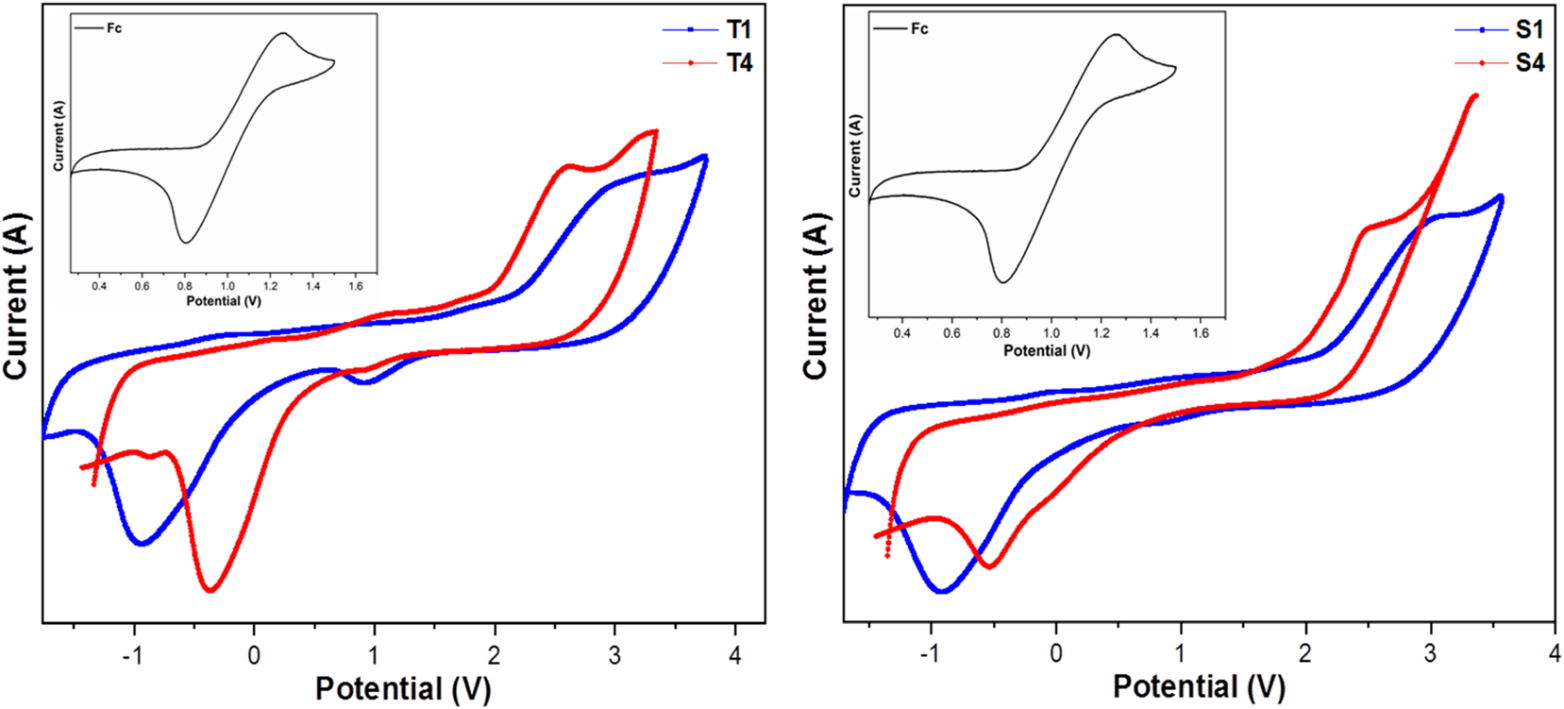
Cyclovoltammograms of lanthanide complexes.

A potential range of −2 V to +4 V and a scanning frequency of 0.1 V s^−1^ are often used to record cyclovoltammograms (CV). 0.1 M tetrabutylammonium perchlorate in DCM was used as supporting electrolyte and silver wire as a reference electrode. The concentration of ferrocene and the complexes were 10^−3^ M. CV of synthesized complexes (T1, T4, S1 and S4) with ferrocene in inset is shown in Fig. 5. As the working electrode and counter electrode, respectively, we have chosen glassy carbon (C) and platinum wire. According to eqn (2) and (3), the energy was measure for HOMO and LUMO.^49,50^

2 *E*_HOMO_ = −[(*E*_ox_) + 3.77] eV

3 *E*_LUMO_ = −[(*E*_red_) + 3.77] eV

Ferrocene (Fc) has a half potential of 1.03 V.^51^Table 4 lists the electro-chemical parameters of prepared complexes. The electronic band gap of complexes is in the category of semiconductors, implying their conductive nature. Values of electronic band gap determined from electrochemical data corroborate with the optical band gap values obtained from absorption spectral data. The change in values of redox potentials of synthesized complexes from that of heteroaromatic auxiliary units suggests the formation of ternary lanthanide complexes.^29,52^

**Table 4:** Electro-chemical data of complexes (T1, T4, S1 & S4)^a^

Complex	*E*_ox_ (V)	*E*_red_ (V)	*E*_HOMO_ (eV)	*E*_LUMO_ (eV)	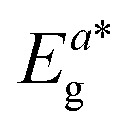 (eV)
T1	2.924	−0.928	−6.694	−2.842	3.852
T4	2.606	−0.357	−6.376	−3.413	2.963
S1	3.013	−0.918	−6.783	−2.852	3.931
S4	2.476	−0.527	−6.246	−3.243	3.003

a
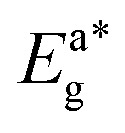
: electronic band gap.

### 3.6 Powder XRD analysis

**Fig. 6:**
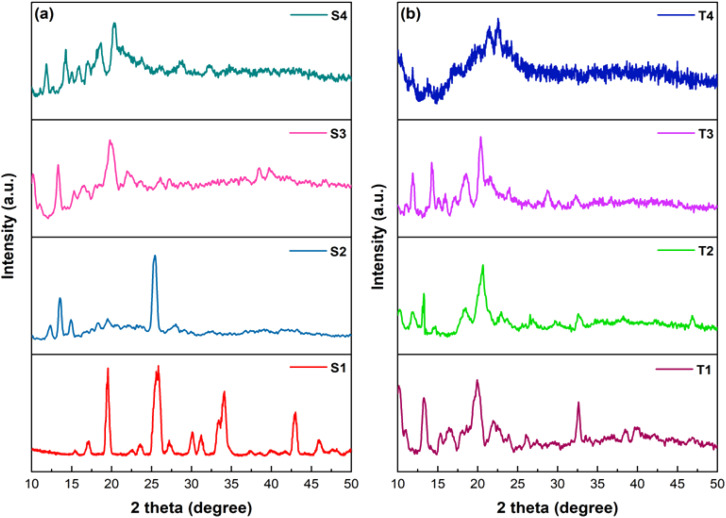
Powder X-ray diffraction (XRD) patterns of S1–S4 (a) and T1–T4 (b) complexes.

To get an idea about crystalline nature of synthesized ternary complexes, their powder X-ray diffraction (XRD) patterns were recorded. Fig. 6(a and b) demonstrates the diffractogramms of lanthanide complexes recorded at Bragg's angle of 2*θ* in range of 10°–50°. The sharp peaks in XRD profiles suggest that the crystalline nature of synthesized complexes. From powder XRD patterns, it can be found that the prepared complexes hold different degree of crystallinity.^53^ High crystallinity degree in ternary Ln(iii) complexes is evidenced by better defined peaks in their diffractogramms.

### 3.7 PL study

**Fig. 7:**
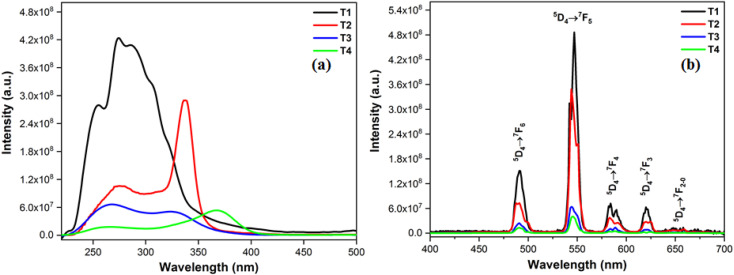
(a) Excitation (at *λ*_em_ = 549) and (b) emission spectral profiles T1–T4.

**Fig. 8:**
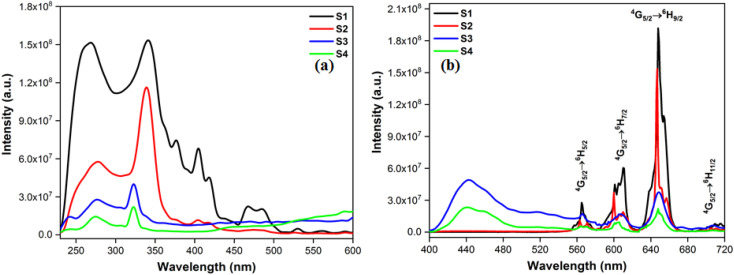
(a) Excitation (at *λ*_em_ = 648) and (b) emission spectral profiles S1–S4.

The photoluminescence profiles of terbium (T1–T4) and samarium (S1–S4) complexes monitored in solid form are demonstrated in Fig. 7 and 8 respectively. Excitation profiles of Tb(iii) complexes evince the broad band of ligand as well as few weak intense peaks owing to *f*–*f* transitions of metal ion. Emission spectral profile of ternary terbium complexes were obtained at their respective excitation wavelength (*λ*_ex_) in solid state. Emission spectra display peaks in 480–620 nm, characteristic of Tb^3+^ ion. The peaks in photoluminescence emission spectra are positioned at 490, 546, 590 and 617 nm attributed to transitions from excited ^5^D_4_ state to lower situated ^7^F_6, 5, 4, 3_ states of Tb^3+^ ion separately.^54,55^ Along with these peaks, the least intense peaks were also seen corresponding to ^5^D_4_ → ^7^F_2–0_. Dominant peak present at around 546 nm is answerable for green emission of T1–T4 complexes.^56^

**Fig. 9:**
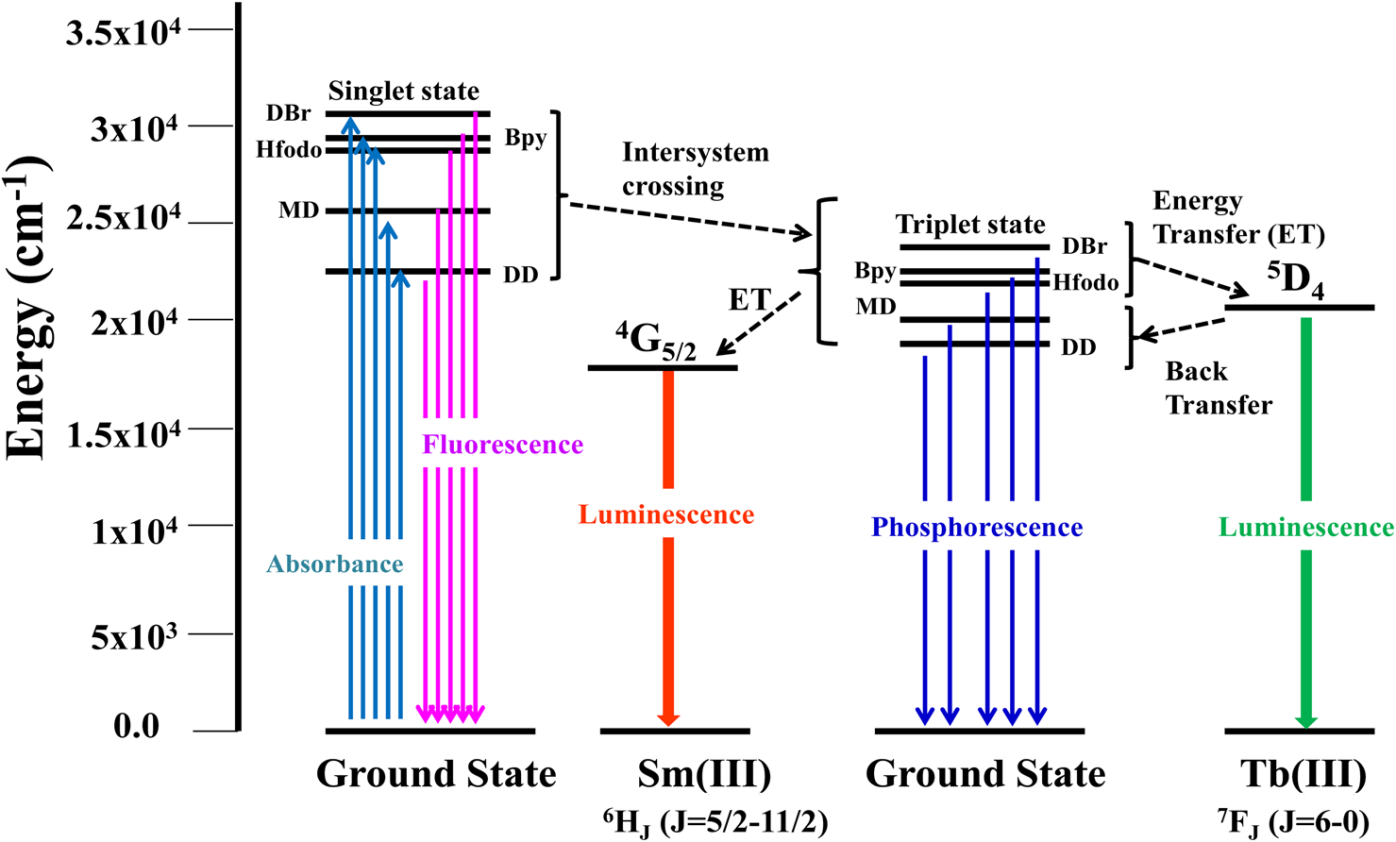
Energy transfer (Antenna Effect) in ternary complexes.

Excitation spectra of S1–S4 evince broad band of ligand as well as few weak intense pinnacles relative to *f*–*f* transitions of Sm^3+^ ion. Emission profiles show characteristic peaks of at 564 nm (^04^G_5/2_ → ^06^H_5/2_), 601 nm (^4^G _5/2_ → ^6^H_7/2_), 648 nm (^4^G_5/2_ → ^6^H_9/2_) and 707 nm (^4^G_5/2_ → ^6^H_11/2_) transition.^57,58^ Ligand dependent bands were appeared in emission spectra of S3 and S4. The emission spectra of S1–S4 evident most pronounced peak at 648 nm depends on the coordinating surrounding of Sm^3+^ ion.^59^ The PL emission intensity decreases in a particular series however it is larger than their binary complex. Hence, the prepared complexes showed enhanced luminescence behavior. The order of emission intensity in synthesized complexes is also supported by energy transfer phenomenon as shown in Fig. 9. Triplet (T) state energy of Hfodo was determined from the phosphorescence spectral profile (Fig. S17†) of its binary complex with Gd(iii) *i.e.* [Gd(Hfodo)_3_(H_2_O)_2_]. The shortest wavelength in the phosphorescence spectrum provides the energy of triplet state of Hfodo. The T level energy of Bpy and its derivatives has already been reported in literature.^60^ Energy transfer parameters of diketone and neutral moieties are listed in Table S1.†eqn (4) was used to estimate the branching ratio (*β*) of synthesized complexes.^61^4
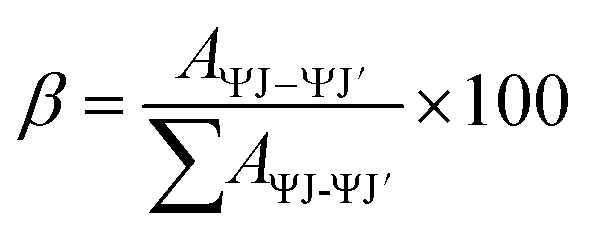
Here, *A*_ψJ–ψ’J_ denotes the integrated area of PL emission peaks. The value of *β* for hyper-sensitive peak is ∼60% as listed in Tables S2† (T1–T4) & S3 (S1–S4), which supports the efficacy of these substances in lasers.^62^ The intensity ratio of ^5^D_4_ → ^7^F_6_/^5^D_4_ → ^7^F_5_ for Tb(iii) and ^4^G_5/2_ → ^6^H_9/2_/^4^G_5/2_ → ^6^H_5/2_ for Sm(iii) demonstrates the asymmetric environment around metal ion.^63^ The quantum yield for synthesized Ln complexes is measured against the reference *i.e.,* quinine bisulphate in dilute sulphuric acid. It is calculated in order to investigate the influence of different neutral moieties on the PL intensity of complexes. Eqn (5) is used for the measurement of relative quantum yield (*Φ*_*s*_).^64^5
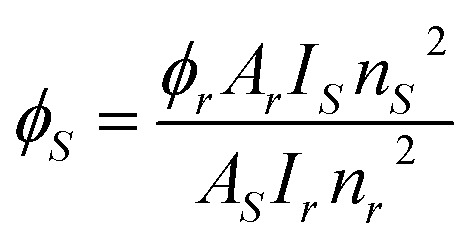
Here, *Φ*_r_, *A*, *I*, *s*, *r* and *n* represent quantum yield reference, absorbance at the excitation wavelength, integrated emission intensity, sample, reference and refractive index of solvent, respectively.^65^ The value of *Φ*_*r*_ is 54.6%. Quantum yield is found to be in the declining manner and support the outcomes obtained from emission spectral profiles. The numerous absorption and photo-physical data are compiled in Table 5.

**Table 5:** Some photo–physical parameters of lanthanide complexes^a^

Complex	*λ*_abs_ (nm)	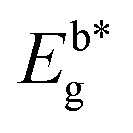 (eV)	*λ*_ex_ (nm)	*λ*_em_ (nm)	FWHM (nm)	Intensity ratio	*Φ*_s_ (%)
T1	281	3.811	274	544	8.69	0.282	6.24
T2	309	3.741	337	547	9.08	0.253	5.67
T3	369	3.101	268	543	10.26	0.357	4.39
T4	382	2.951	369	545	7.95	0.363	3.81
S1	288	3.901	268	648	9.56	10.090	5.13
S2	303	3.819	339	647	8.08	12.388	5.02
S3	373	3.098	323	649	14.08	11.101	2.65
S4	373	3.044	324	648	10.96	9.545	1.76

a
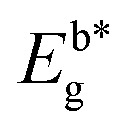
: optical band gap.

### 3.8 Colorimetric study

**Fig. 10:**
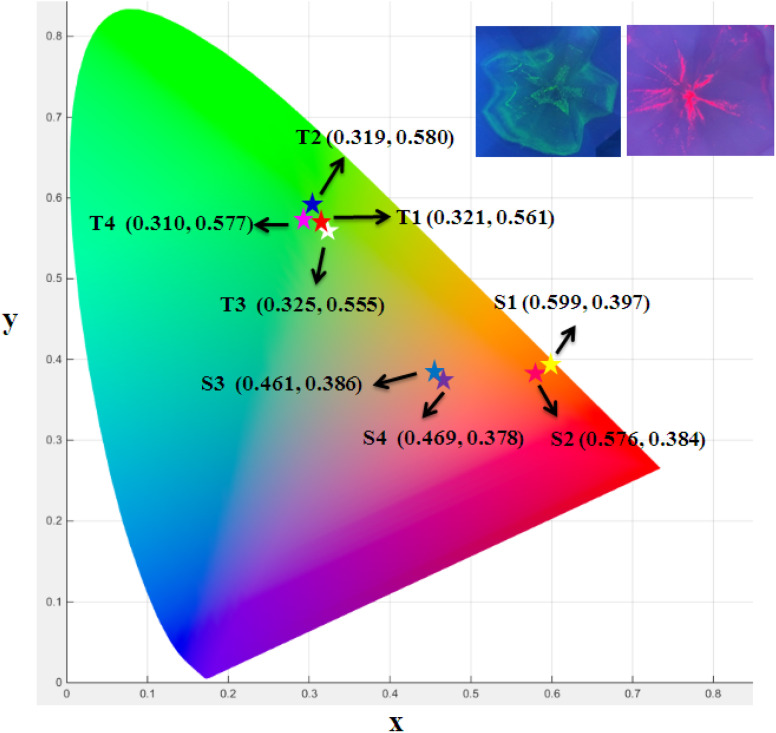
CIE (*x*, *y*) coordinates of prepared complexes (inset figures are fluorescent photographs of T1 and S1).

The color (*x*, *y*) co-ordinates was obtained from emission spectral data. These co-ordinates positioned in the greenish and orange red regime of Fig. 10 relative to T1–T4 and S1–S4, separately. The florescent photographs of T1 and S1 are also shown in CIE (*x*, *y*) diagram of complexes. From (*x*, *y*), the other colorimetric parameters *i.e.,* (*u*′, *v*′) and color temperature (CT) were estimated through eqn (6) and (7).^66–68^ (*u*′, *v*′) with CT values are manifested in Fig. 11.6
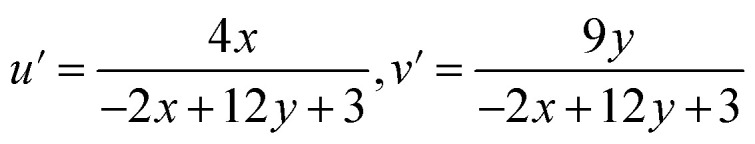

7 CT = −437*n*^3^ + 3601*n*^2^ − 6861*n* + 5514.31

**Fig. 11:**
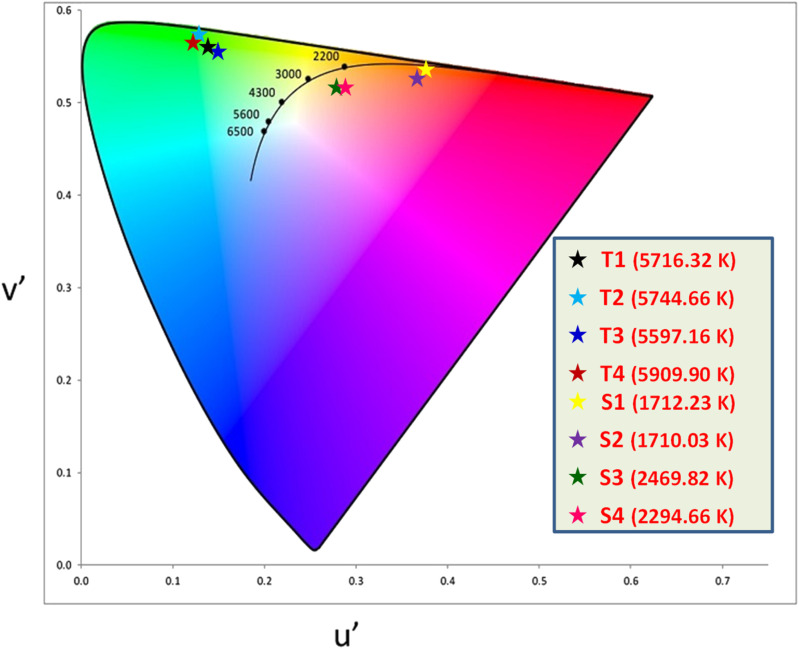
CIE (*u*′, *v*′) parameters of prepared complexes.

In the relation (7), *n* characterizes the inverse-slope line and was assessed *via*eqn (8).8
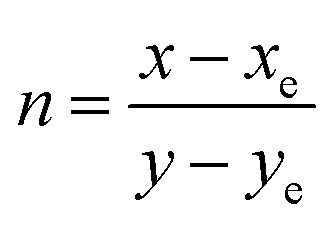
Here, *x*_e_ and *y*_e_ represents the color epicenter having respective entity *i.e.* 0.332 and 0.186. Color purity (CP) of prepared Ln complexes was determined from eqn (9).9
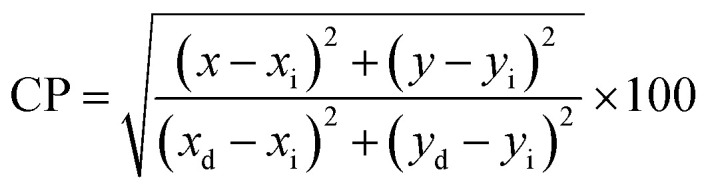
Here, (*x*_i_ & *y*_i_) with value 0.333 are the illuminated points. The value of dominant points *x*_d_ = 0.290 (green) & 0.688 (red) and *y*_d_ = 0.600 (green) & 0.331 (red).^69–71^ The relative luminance value for a certain color in terms of RGB parameters is also calculated. The color characteristics for ternary lanthanide complexes are listed in Table 6.

**Table 6:** Colorimetric parameters of prepared complexes

Complex	(*x*, *y*)	(*u*′, *v*′)	CP (%)	*R*	*G*	*B*	Luminance (%)
T1	(0.321, 0.561)	(0.141, 0.555)	84.42	110	254	57	82
T2	(0.319, 0.580)	(0.137, 0.559)	91.48	96	254	28	81
T3	(0.325, 0.555)	(0.144, 0.554)	82.14	120	255	61	83
T4	(0.310, 0.577)	(0.133, 0.558)	90.62	79	255	46	81
S1	(0.599, 0.397)	(0.365, 0.544)	77.07	255	98	0	60
S2	(0.576, 0.384)	(0.359, 0.535)	69.94	255	101	0	61
S3	(0.461, 0.386)	(0.275, 0.518)	39.02	255	157	101	72
S4	(0.469, 0.378)	(0.284, 0.516)	40.35	255	148	100	70

## 4 Conclusions

A different series of Ln complexes with fluorinated primary and heteroaromatic secondary ligand have been prepared and examined. The outcome of infrared study reveals the coordination of ligands through hard donor atoms (–O and –N). The complexes exhibit band at higher wavelength as compared to Hfodo which illustrates the stability of ligand orbitals on complexation. The high luminescent intensity recommends the superior ligand sensitization for solid sample. Synthesized terbium and samarium complexes emit bright green and orange red emission which is the constituent of tricolor system. The prepared lanthanide complexes are potential applicant in laser diodes due to high *β* value corresponding to the hyper-sensitive transition and the range of band gap further prove their utility as conducting material in fabricating displays.

## Ethical statement

The article does not involve any study performed on animals or human by any of the authors.

## Data availability

The authors affirm that the information/data of this research article is available inside the article.

## Author contributions

Anjli Hooda = data curation, writing – original draft; Devender Singh = writing – review & editing, supervision; Anuj Dalal = investigation; Kapeesha Nehra = formal analysis; Sumit Kumar = visualization; Rajender Singh Malik = software; Ramesh Kumar = methodology; Parvin Kumar = resources; Brijesh Rathi = validation.

## Conflicts of interest

The authors declare that they have no known competing financial interests or personal relationships that could have appeared to influence the work reported in this paper.

## Supplementary Material

RA-013-D3RA00214D-s001
