# Supplementary material for: Optical, electrochemical and photophysical analyses of heteroleptic luminescent Ln(iii) complexes for lighting applications
Source: RSC Adv. 2023 Mar 20;13(13):9033–45. doi: 10.1039/d3ra00214d (PMC10025952; doi:10.1039/d3ra00214d)
Supplement: RA-013-D3RA00214D-s001 [file RA-013-D3RA00214D-s001.pdf]

Electronic Supplementary Material (ESI) for RSC Advances.
This journal is © The Royal Society of Chemistry 2023

Optical, Electrochemical and Photophysical Analyses of Heteroleptic Luminescent Ln(III) Complexes for Lighting Applications

Anjli Hooda[a], Devender Singh[a*], Anuj Dalal[a], Kapeesha Nehra[a], Sumit Kumar[b], Rajender Singh Malik[b], Ramesh Kumar[c] and Parvin Kumar[c]

[a]*Department of Chemistry, Maharshi Dayanand University, Rohtak-124001, India.*
[b]*Department of Chemistry, DCR University of Science & Technology, Murthal, Haryana 131039, India.*
[c]*Department of Chemistry, Kurukshetra University Kurukshetra, -136119, Haryana, India.*

Electronic Supporting Information

Table S1 Energy transfer parameters of Hfodo and substituted 2,2′-bipyridine derivatives

Ligand	E_{S1}	E_{T1}	$\Delta E_{(S1\text{-}T1)}$	$\Delta E_{(T1\text{-}5D4\ of\ Tb3+)}$	$\Delta E_{(T1\text{-}4G5/2\ of\ Sm3+)}$
**Hfodo**	29,154	22,500	6,654	2,134	4,833
**Bpy**	29,673	22,883	6,790	2,517	5,216
**DBr**	31,152	23,696	7,456	3,330	6,029
**MD**	25,706	20,161	5,545	-205	2,494
**DD**	22,727	18,315	4,412	-2051	648

Table S2 Percentage branching ratio of various transitions involved in T1-T4

Complex	Percentage branching ratio of transitions					
	Ligand	$^5D_4\rightarrow^7F_6$	$^5D_4\rightarrow^7F_5$	$^5D_4\rightarrow^7F_4$	$^5D_4\rightarrow^7F_3$	$^5D_4\rightarrow^7F_{2,1,0}$
**T1**	-	18.06	64.09	10.94	6.26	0.64
**T2**	-	17.35	68.63	8.29	5.32	0.40
**T3**	-	21.40	59.92	12.11	6.21	0.35
**T4**	-	23.19	63.90	8.69	3.77	0.44

Table S3 Percentage branching ratio of various transitions involved in S1-S4

Complex	Percentage branching ratio of transitions				
	Ligand	$^4G_{5/2}\rightarrow^6H_{5/2}$	$^4G_{5/2}\rightarrow^6H_{7/2}$	$^4G_{5/2}\rightarrow^6H_{9/2}$	$^4G_{5/2}\rightarrow^6H_{11/2}$
**S1**	-	6.52	24.91	65.79	2.77
**S2**	-	2.28	21.45	75.41	0.01
**S3**	66.74	1.97	7.35	21.87	2.06
**S4**	67.96	2.35	6.69	22.43	0.06

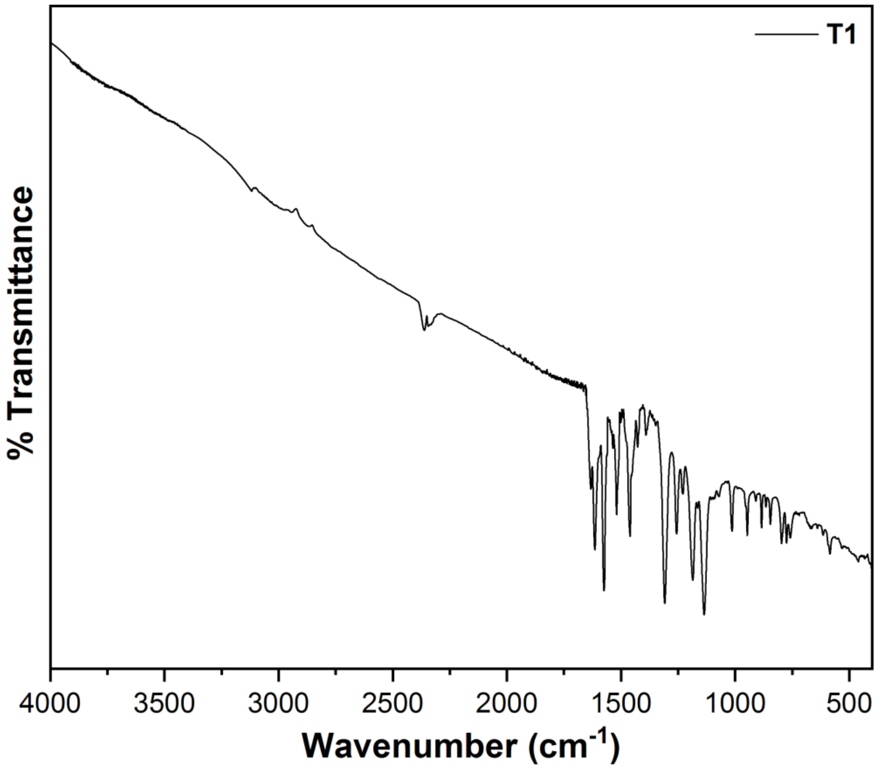

Fig. S1 IR spectrum of T1

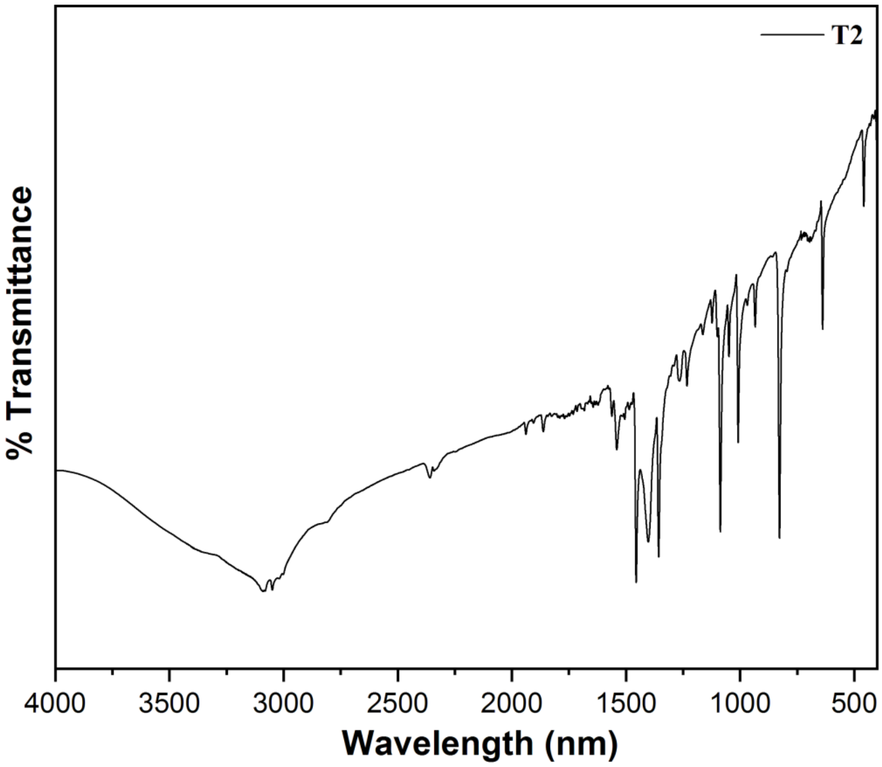

Fig. S2 IR spectrum of T2

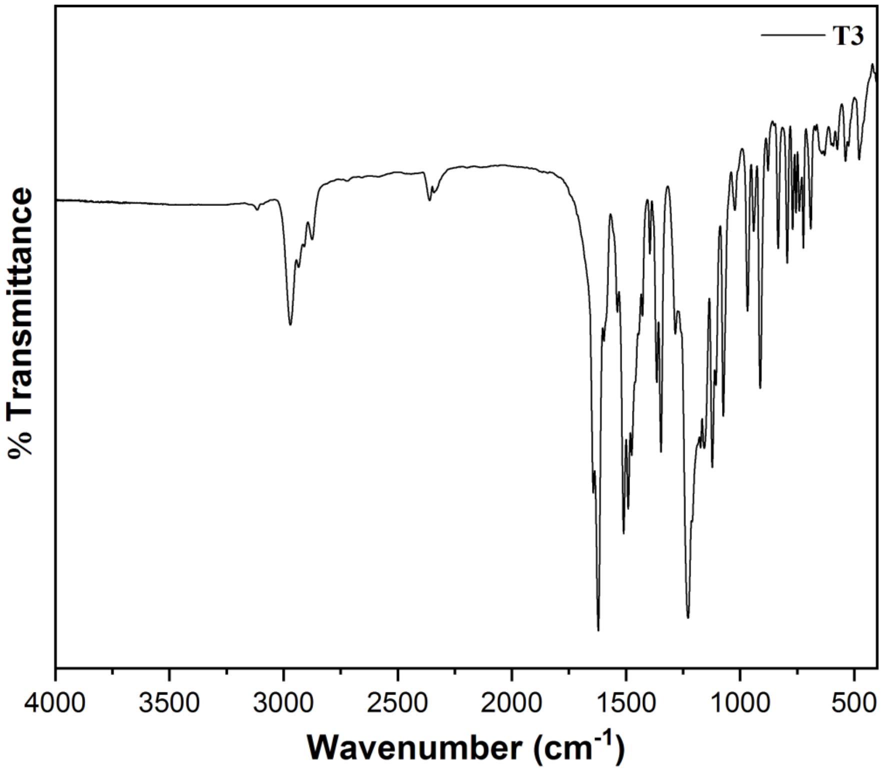

Fig. S3 IR spectrum of T3

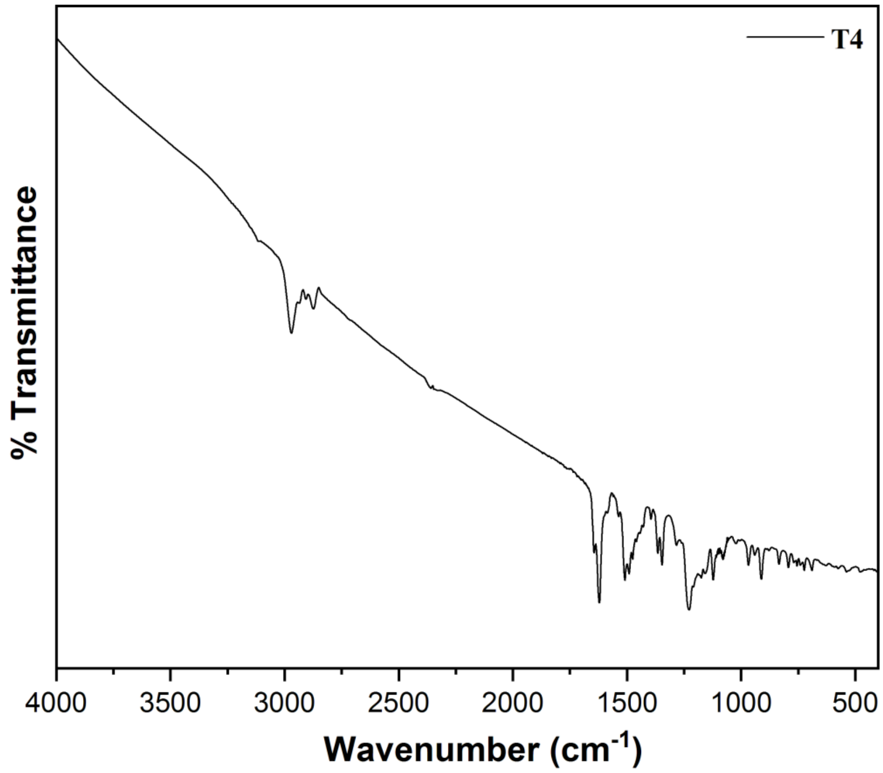

Fig. S4 IR spectrum of T4

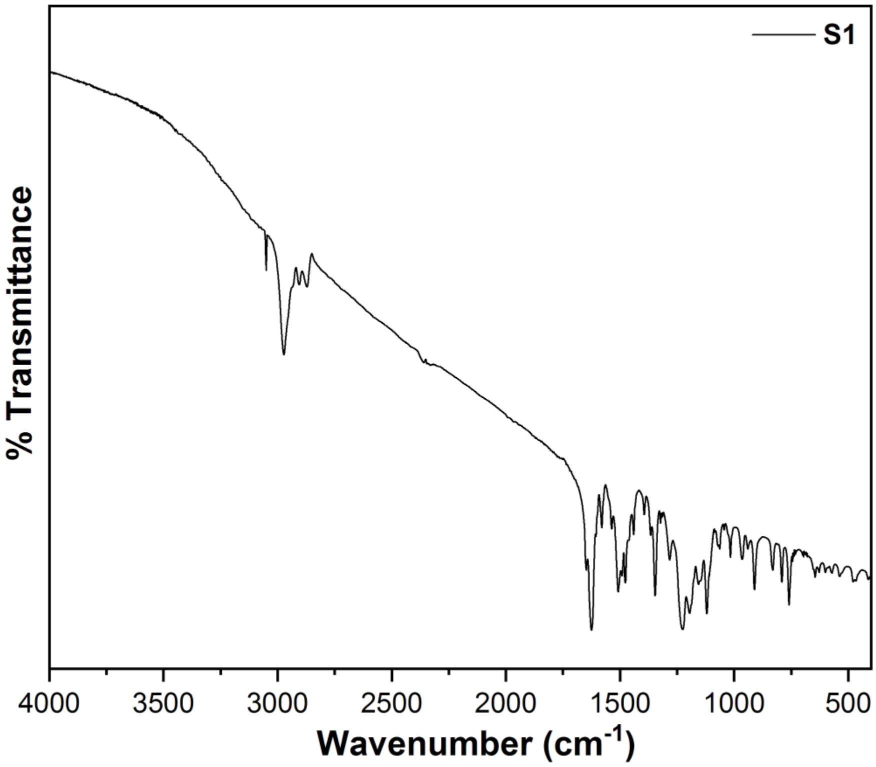

Fig. S5 IR spectrum of S1

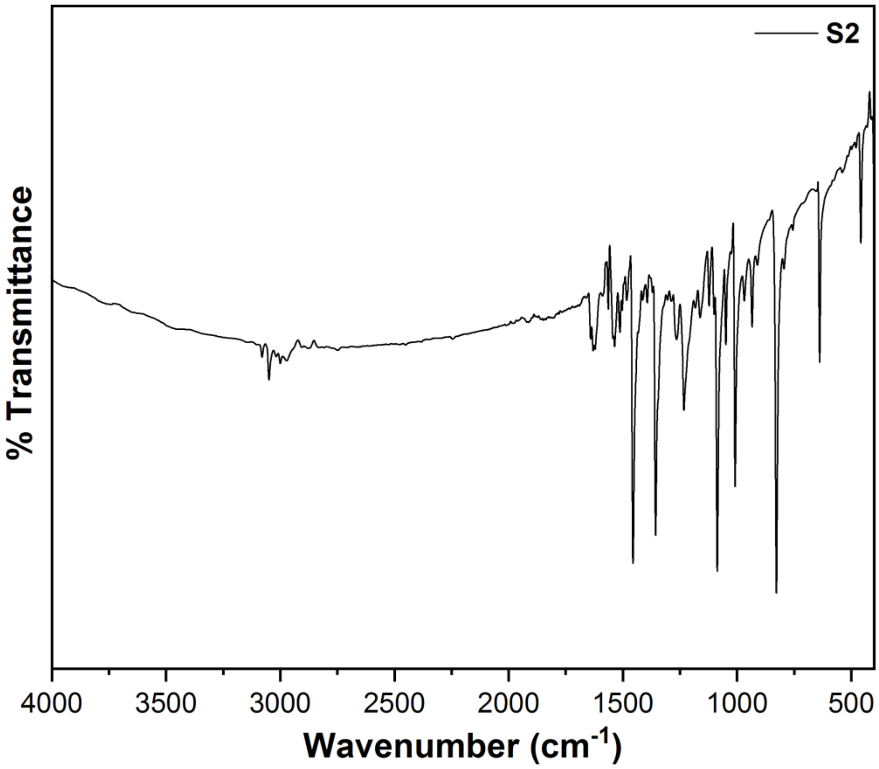

Fig. S6 IR spectrum of S2

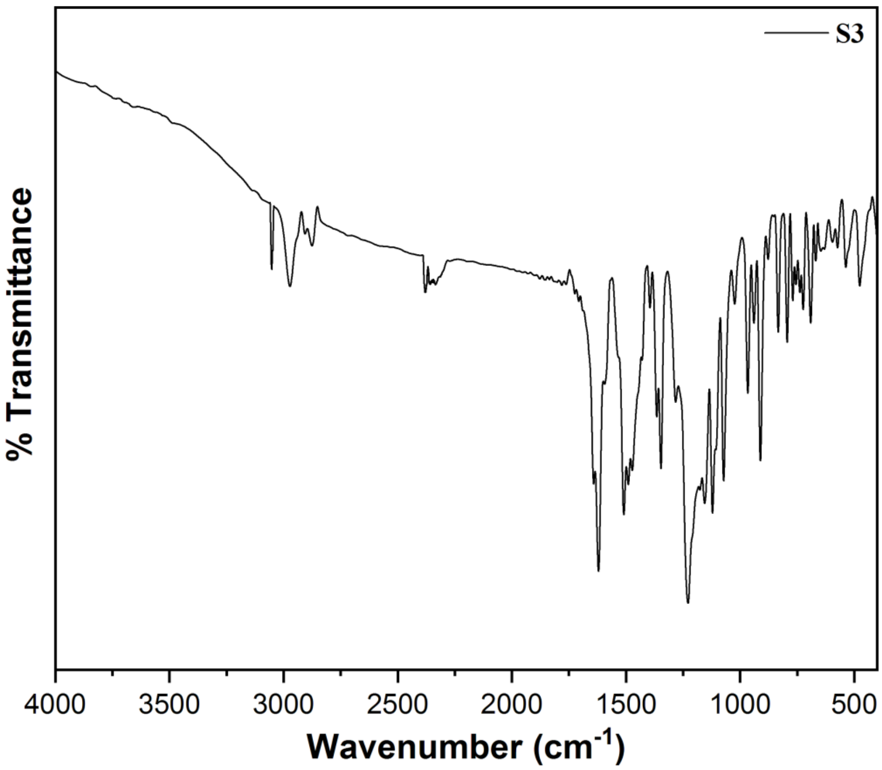

Fig. S7 IR spectrum of S3

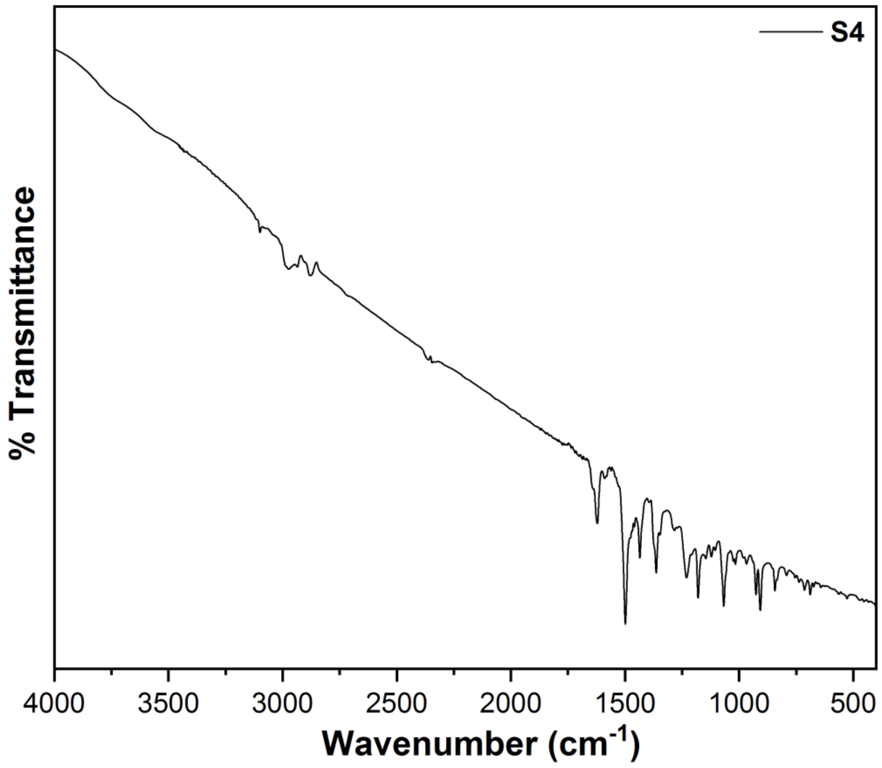

Fig. S8 IR spectrum of S4

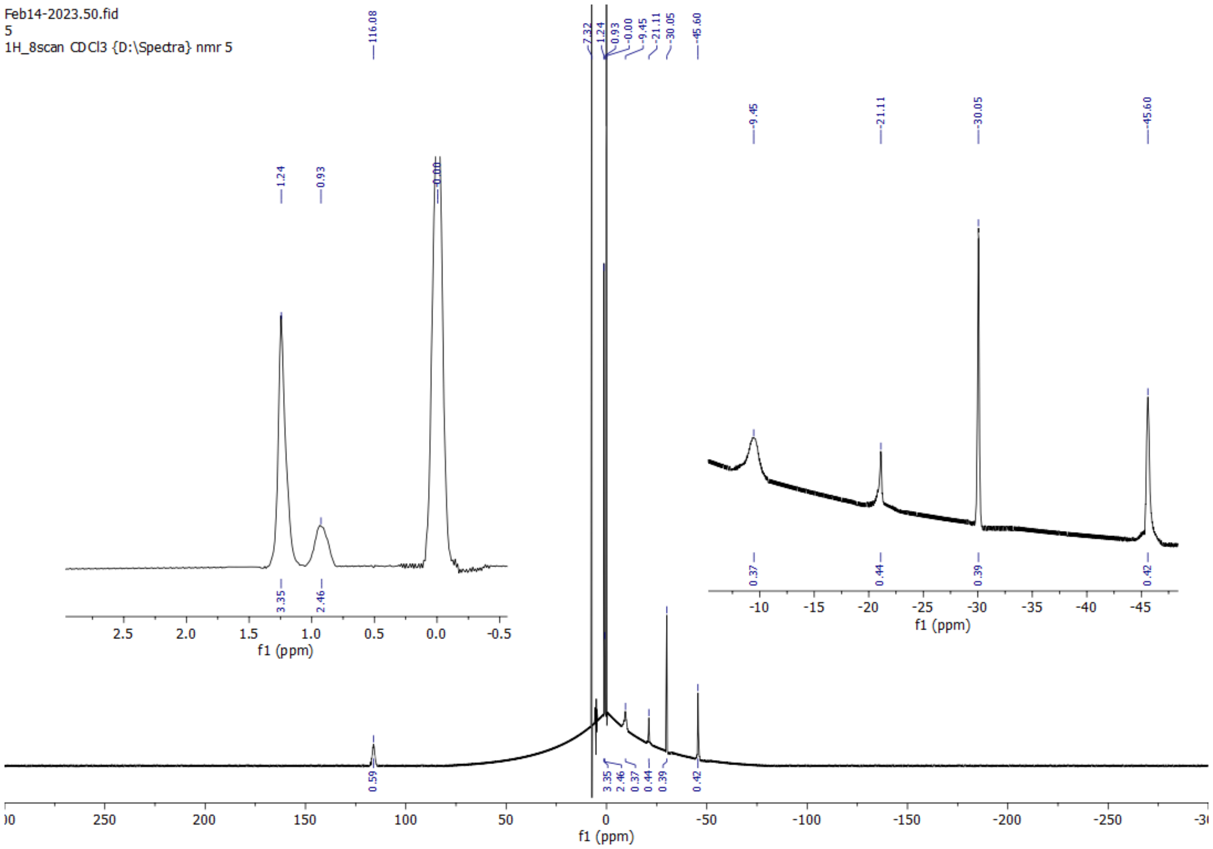

Fig. S9 NMR spectrum of T1

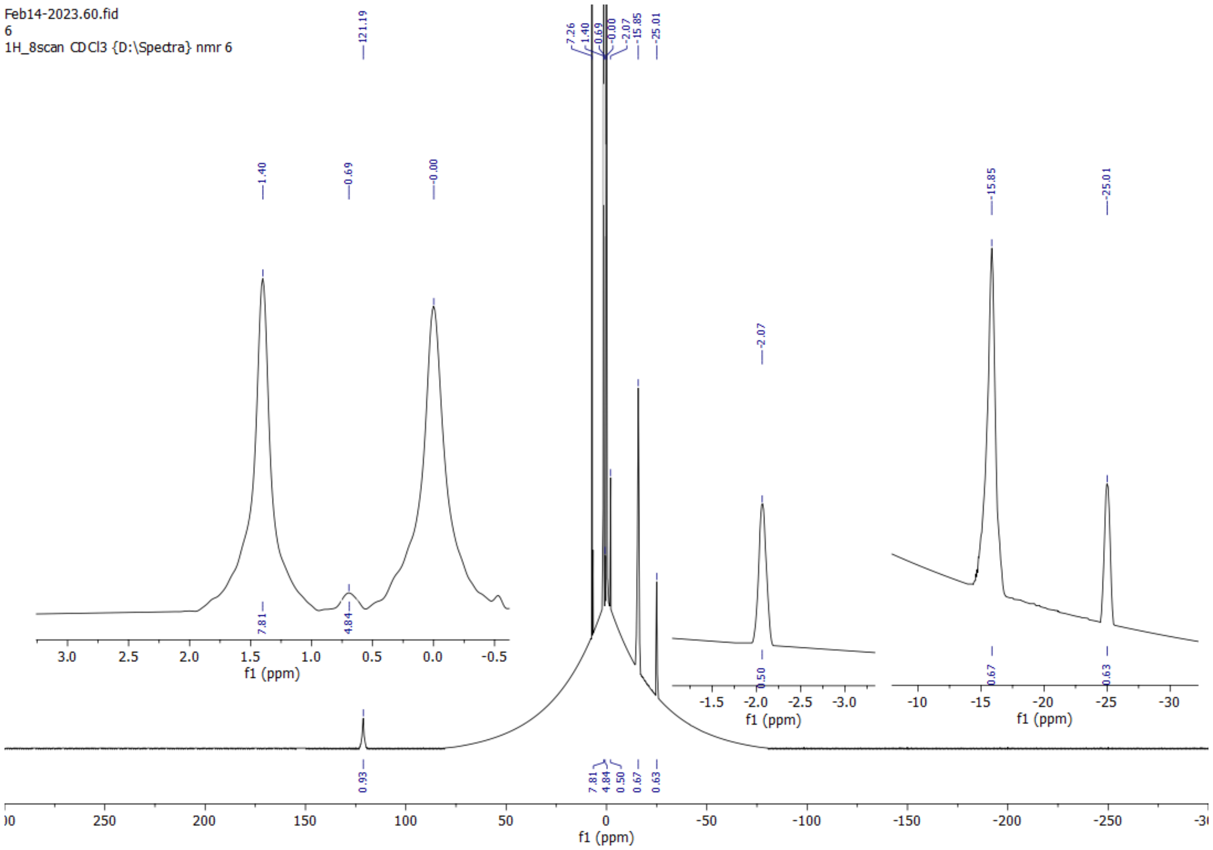

Fig. S10 NMR spectrum of T2

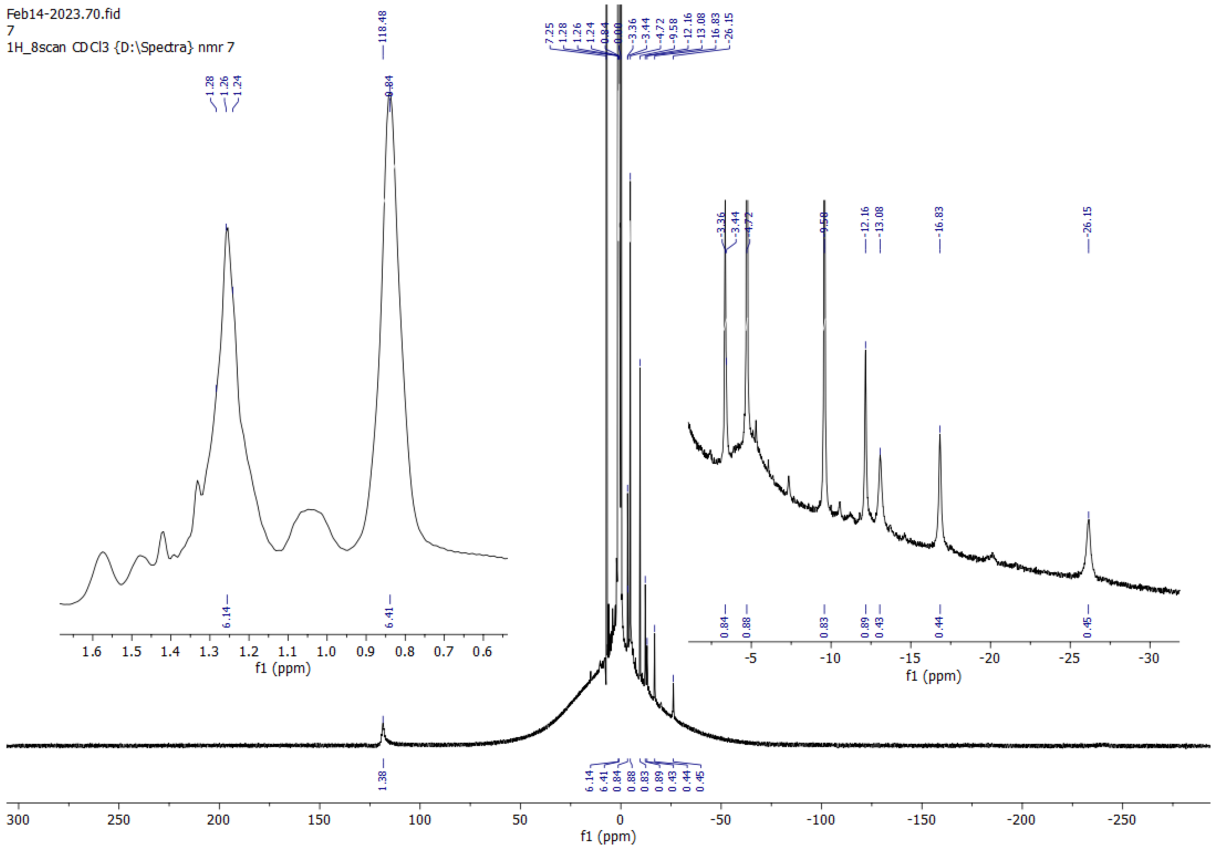

Fig. S11 NMR spectrum of T3

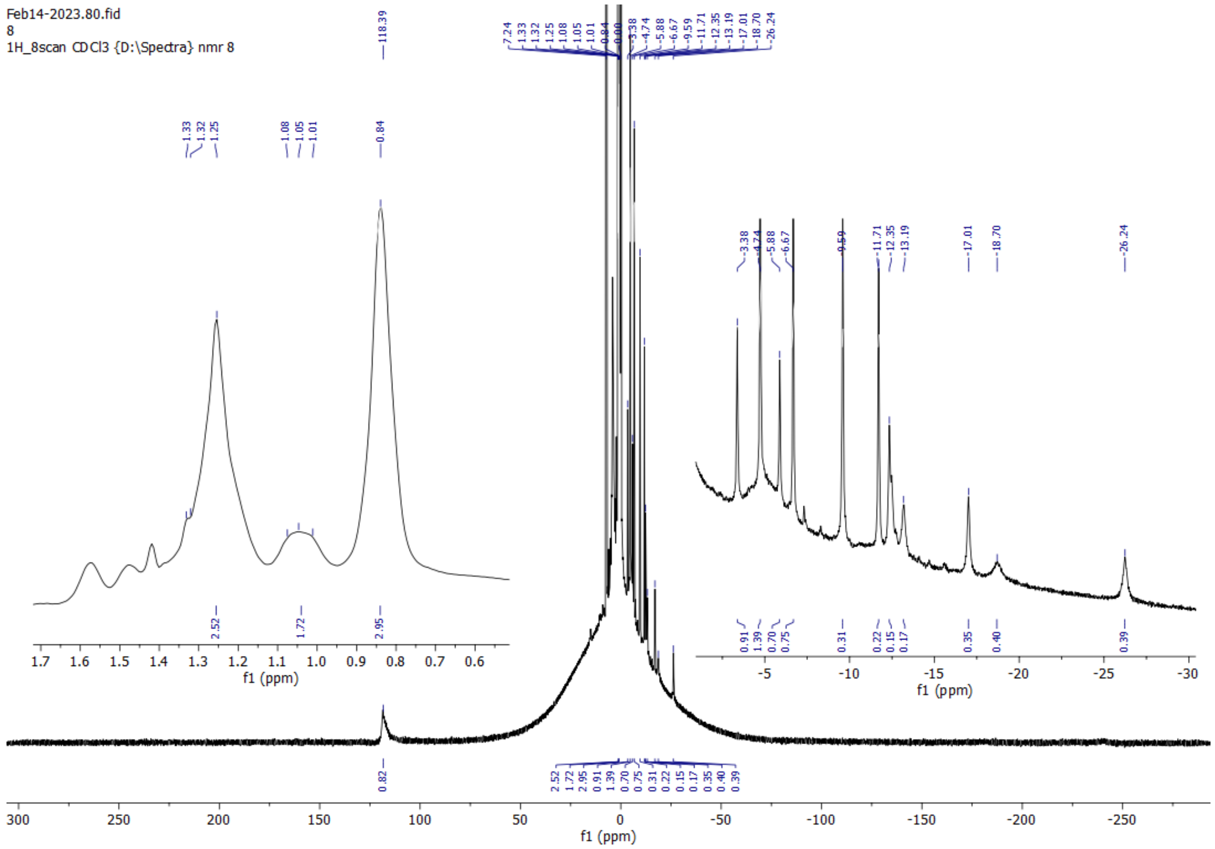

Fig. S12 NMR spectrum of T4

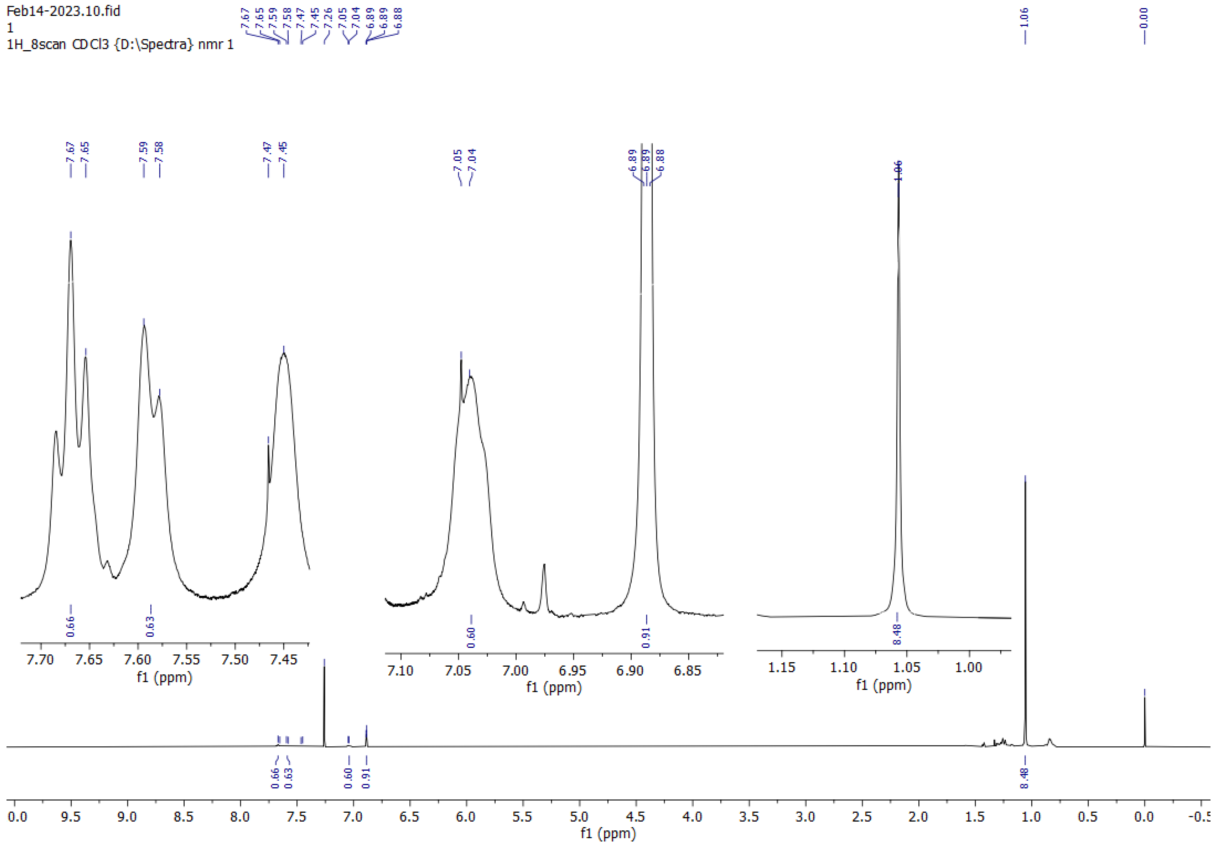

Fig. S13 NMR spectrum of S1

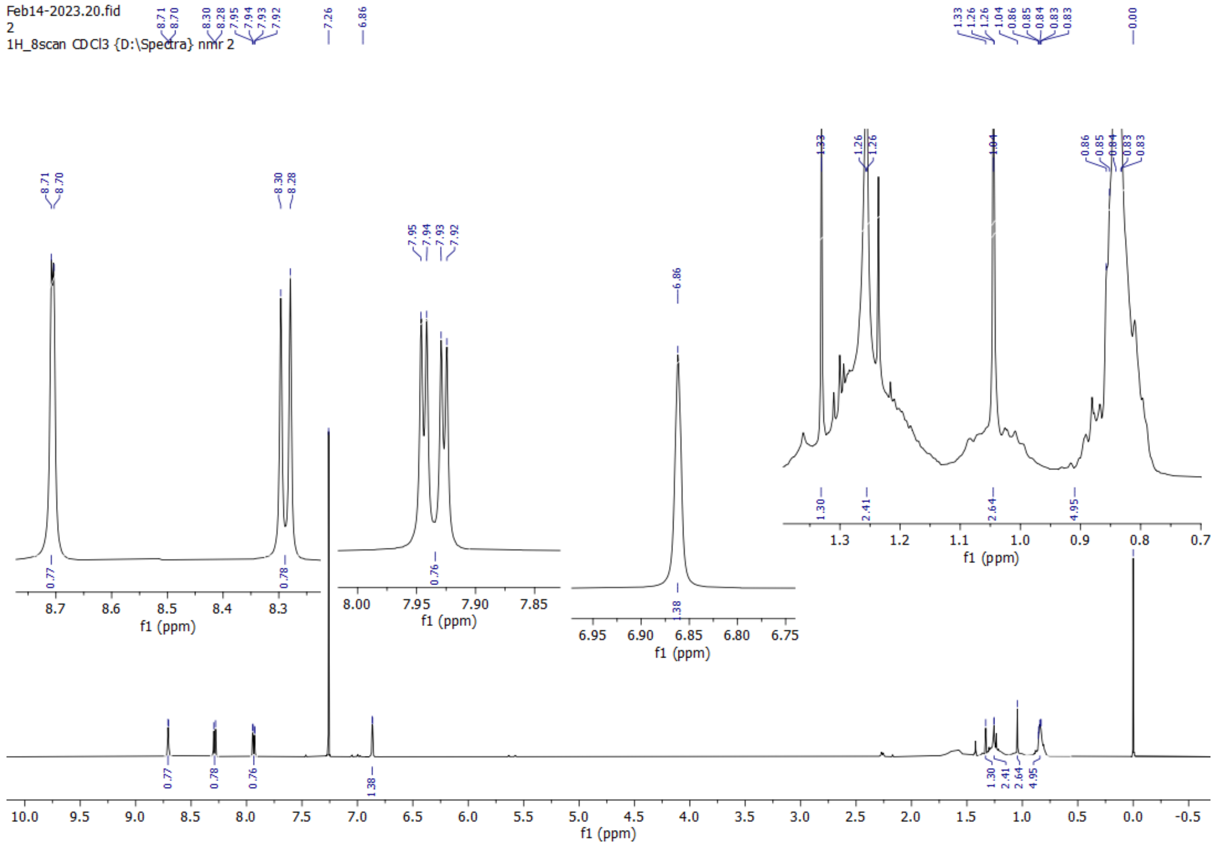

Fig. S14 NMR spectrum of S2

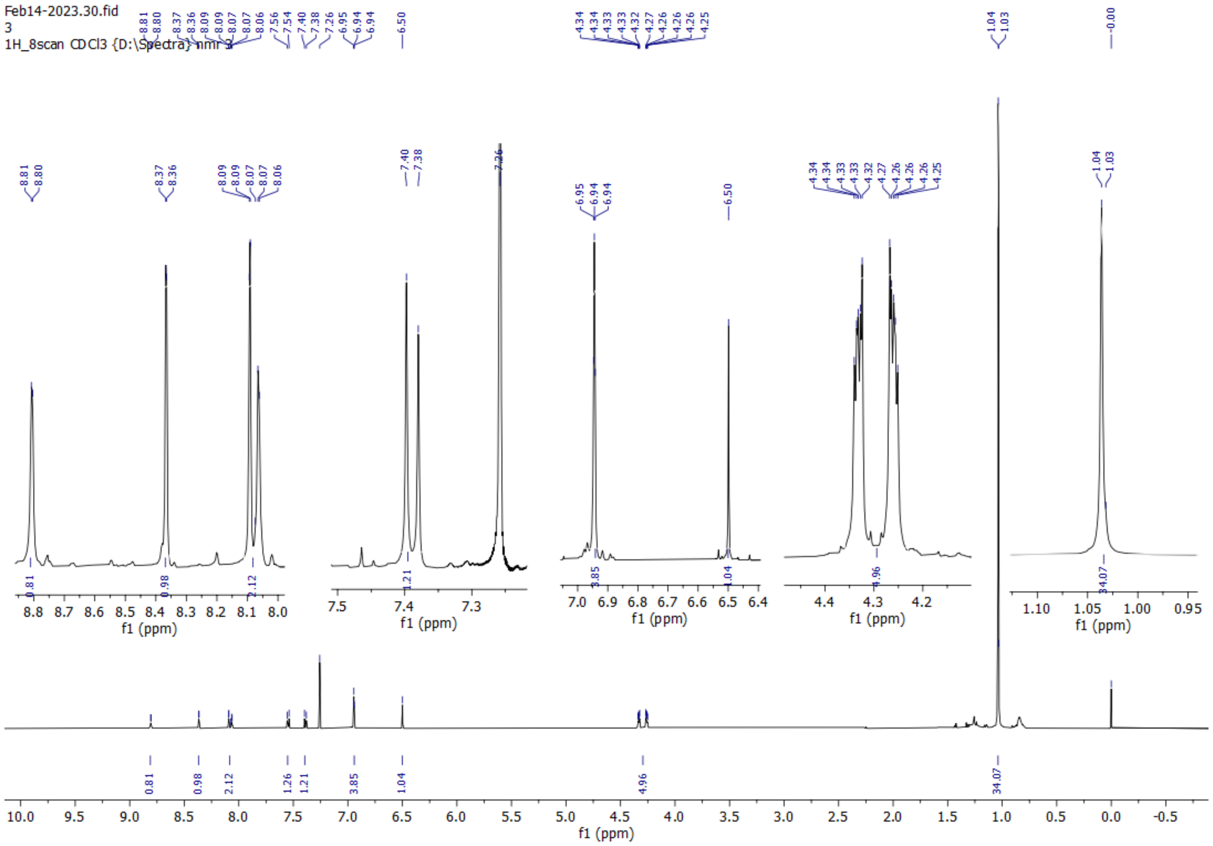

Fig. S15 NMR spectrum of S3

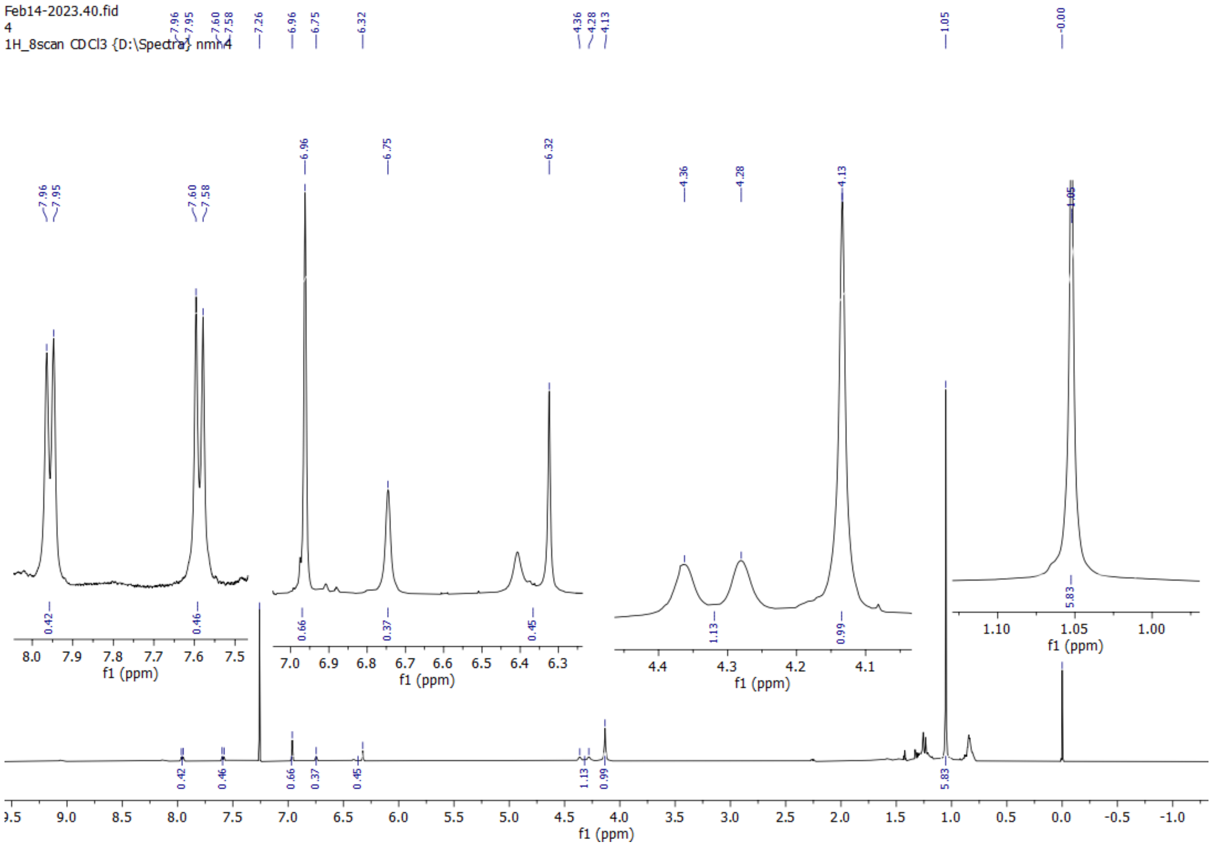

Fig. S16 NMR spectrum of S4

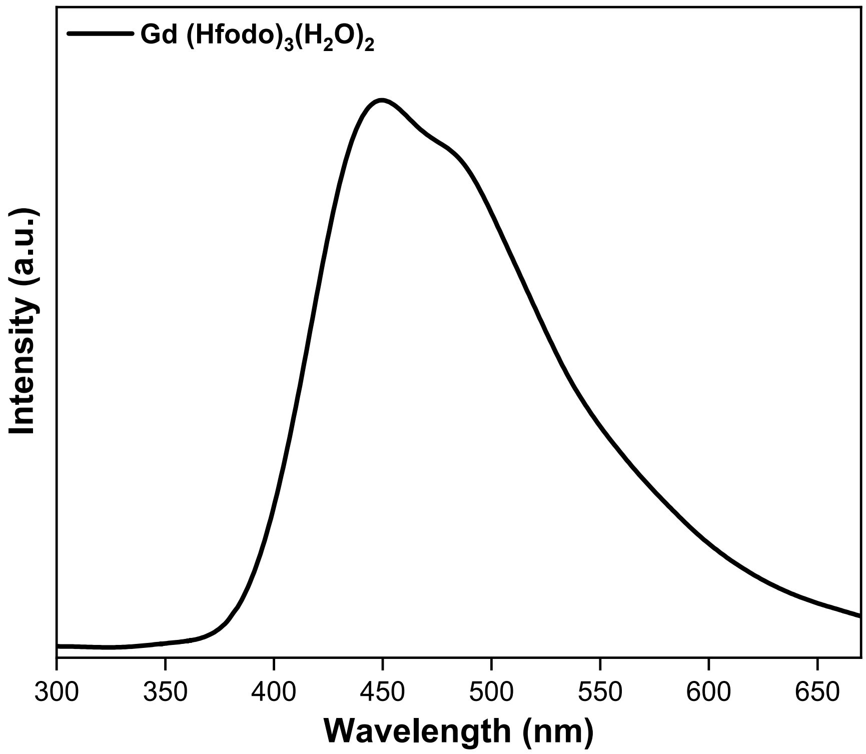

Fig. S17 Phosphorescence spectrum of $[Gd(Hfodo)_3(H_2O)_2]$
